# Exploring the feasibility and effectiveness of a naturalistic family centered intervention to enhance early interactions in toddlers with Down syndrome

**DOI:** 10.1038/s41598-025-96803-z

**Published:** 2025-04-09

**Authors:** Ana Mendoza-García, Andrés Aparicio, Paulina Sofía Arango, Marcela Tenorio

**Affiliations:** 1 Departamento Interfacultativo de Psicología Evolutiva y de la Educación, Universidad Autónoma de Madrid, Madrid, Spain; 2 Millennium Institute for Care Research (MICARE), Santiago, Chile; 3 Centro de Investigación para la Mejora de los Aprendizajes, Facultad de Educación, Universidad del Desarrollo, Santiago, Chile

**Keywords:** Intervention, Down syndrome, Early interactions, Sensitive responsiveness, Directiveness, Psychology, Human behaviour

## Abstract

This study analyses the feasibility and effectiveness of BabyMICARE, a manualised intervention programme aimed at improving the interactions between caregivers and infants and toddlers with Down syndrome. The programme’s goal is to enhance caregivers’ sensitivity and reduce directivity during early interactions, particularly during play and daily routines. A pre-test and post-test design was used with 40 dyads of infants with Down syndrome and their caregivers, who were divided into a control group (n = 20) and an intervention group (n = 20), based on baseline scores in key interaction subscales. Sessions were conducted over 10 weeks by trained psychologists. Parent-infant interaction was assessed using the MACI coding system, which measures aspects such as sensitive responsiveness, directivity and the level of reciprocity between the parent and the child. The programme showed high feasibility, with a 100% attendance rate but some rescheduling. Caregivers evaluated it positively. The intervention group demonstrated significant improvements in five of eight MACI scales, particularly in sensitive responsiveness and nondirectiveness, while no changes were observed in the control group. The results suggest that BabyMICARE is a feasible and effective intervention for promoting more responsive, less directive interactions, which may be crucial in fostering children’s development.

## Introduction

Down syndrome is a genetic condition characterised by the complete or partial presence of an extra chromosome in the 21st chromosome^1^. It is the most common aneuploidy in the population worldwide, with an estimated incidence of 1 in 1000 live births^2^. Furthermore, in Latin America, Chile is the country with the highest prevalence rate (24.7 per 10,000 births)^3,4^

While there is high intrasyndromic variability, some atypical trajectories that appear to be common in the development of children with Down syndrome have been described in the scientific literature^5^. Individuals with this condition have increased vulnerability to developing intellectual disability, a neurodevelopmental condition characterised by significant limitations in intellectual functioning and adaptive behaviour that are evident in the developmental period^6–8^. Adaptive behaviour refers to a set of practical, social and conceptual skills that people learn and implement to function in their daily routines^9^. Its acquisition and development are essential to support the social participation of people with intellectual disabilities and to determine their support needs^10^. Moreover, since adaptive behaviours are learned, it is expected that practice and appropriate support from the environment effectively stimulate these skills^11^.

Research in developmental psychology has shown that social interactions in early childhood are fundamental for development^12^. Early interactions are defined as communicative and affective exchanges that occur from the first years of life between adults and infants^13,14^. In these early encounters, infants develop and acquire essential skills for effective social participation, such as the development of communication^15,16^ and adaptive behaviours that allow them to gain agency and independence in their daily routines^11,17^. In this context, the use of external world referents (objects, toys, tools, among others) serves not only to stimulate and promote play with the youngest children but also to progressively integrate them into the social and cultural routines of their environment^18,19^.

In early childhood, especially before children begin to speak, interpreting the communicative signals emitted by infants can be a complex task, making it challenging for caregivers to provide appropriate and stimulating responses^20^. This difficulty is further heightened in children with atypical developmental trajectories, such as those with Down syndrome, who often experience delays in developing oral language or may not develop it at all^17,21^. These communication and language difficulties complicate caregivers’ ability to interpret and respond effectively to children’s signals, adding an additional layer of complexity to their interactions^22^.

Various constructs have been studied to understand parent–child interactions and their impact on child development, including sensitivity, responsiveness, directiveness, and reciprocity. These constructs are interrelated; for example, sensitivity— defined as the caregiver’s ability to perceive and respond to the child’s cues appropriately^23^—is fundamental for responsiveness, as without sensitivity, it is not possible to respond appropriately. Responsiveness, in turn, ensures appropriate, contingent, supportive reactions, fostering secure attachment and a base for exploration^24^. Directiveness refers to how much the caregiver guides the child’s attentional focus or behavior^25,26^. Reciprocity is the bidirectional exchange between caregiver and child^27^, that has been operationalized as the synchronized and dynamic adaptation of the temporal structure of behaviors and shared affect between interactive partners^28^.

These behaviors are vital for achieving children’s developmental milestones. Sensitivity and responsiveness support emotional regulation and exploration, while balanced non-intrusive directiveness aids skill development. Reciprocal interactions enhance social and cognitive skills, which are crucial for long-term developmental success^29^. Research shows these skills are linked to better attachment, social-emotional competence, and language development^30,31^, making interventions targeting these behaviors important, especially for at-risk populations.

Preliminary studies have documented characteristics associated with early interactions when the dyad includes an infant with Down syndrome. There is a consensus on higher levels of directiveness of mothers of children with Down syndrome than mothers of typically developing infants^32^. Regarding the sensitivity shown in the interaction, some studies report similar sensitivity between mothers of children with Down syndrome and mothers of typically developing children^33,34^. With respect to children, some studies have shown that children with Down syndrome have difficulty directing their attention to caregivers, which makes interaction difficult^35^. There are scarce studies on the interaction itself, but the limited evidence suggests that there is less mutuality and engagement in dyads that include infants with Down syndrome^32^.

On the other hand, it is also relevant to consider the child’s intervention in the interaction by observing behaviours such as attention, bodily emotional expression or activity level, which in turn serve as a guide for the adult’s behaviour. The degree of reciprocity between both participants, adults and infants, can be assessed through elements such as episodes of joint attention, body orientation and shared enjoyment in play, among others^36,37^.

Considering what has been observed when studying parental variables in early interactions, different interventions have been designed over the last few decades, aimed at accompanying caregivers in the challenges of early parenting. The effectiveness of these interventions has been supported by several studies and meta-analyses^30,38^. Research shows that working with the caregiver has a more significant impact than working directly with the child, as it is the adult who guides, cares for and accompanies the child in their development and learning^39^. For this reason, interventions usually focus on developing important skills for caregivers, such as sensitivity, which allows them to detect, interpret and respond appropriately to children’s signals^40–42^.

Effective intervention programs for promoting parental sensitivity share several key characteristics. They focus on enhancing real-time caregiver-child interactions by providing actionable strategies to help caregivers respond appropriately to their child’s signals, thereby strengthening the caregiver-child bond and supporting socioemotional or socio-communicative development^30,43–48^. Immediate feedback, often delivered through live coaching or video reviews, enables caregivers to reflect on and adjust their behaviors in the moment, significantly improving sensitivity^31^.

These programs are typically brief and targeted, concentrating on specific skills to avoid overwhelming caregivers while promoting gradual behavioral change^43^. They incorporate task-specific support, modeling, practice, continuous feedback, and video demonstrations, often delivered in natural environments like the home^49^. Structured opportunities for practice and repetition facilitate the internalization and generalization of these skills into everyday interactions, while flexibility and cultural responsiveness ensure better outcomes by addressing families’ unique needs.

Furthermore, successful interventions prioritize building caregiver confidence and empowerment by emphasizing their critical role in their child’s development and fostering their ability to engage in supportive interactions. Despite the effectiveness of these approaches, no existing programs in Latin America focus specifically on infants and toddlers (0–3 years) with developmental risk in play contexts and daily routines. The local development of BabyMICARE ensures cultural relevance, addressing the specific social and familial contexts of Latin America while filling gaps in early intervention services often lacking in public systems. To address this gap, we developed an evidence-based program tailored to meet these criteria.

The aim of this report is to present the feasibility and efficacy analysis of BabyMICARE, an evidence-based, manualized program designed to enhance parenting skills and improve observed sensitive responsiveness and nondirectiveness in early caregiver-infant interactions, specifically for families of infants with Down syndrome.

The development of BabyMICARE draws from several evidence-based early intervention programs that have demonstrated effectiveness in improving caregiver-child interactions, a cornerstone of child development. These programs, including Attachment and Biobehavioral Catch-up (ABC)^50^ and Promoting First Relationships (PFR)^44^, employ strategies such as real-time feedback and video-based interventions to enhance parental sensitivity, attachment security, and understanding of children’s emotional needs. Additional influences, such as the Family Guided Routine-Based Intervention (FGRBI)^48^, BabyUbuntu (n.d.), and the WHO’s Caregiver Skills Training (CST)^46^, emphasize naturalistic, culturally responsive strategies and support caregiver empowerment to promote engagement, communication, and inclusion in everyday activities.

Unlike programs designed for typically developing children, some of these interventions address the unique challenges and needs associated with developmental conditions^46,51^. They prioritize tailored approaches that equip caregivers with the skills and confidence to navigate specific interaction patterns and developmental trajectories. Moreover, they integrate strategies into daily routines, fostering inclusion and aligning interventions with the diverse cultural and contextual realities of families facing developmental vulnerabilities.

The BabyMICARE programme was developed in Chile for infants and toddlers aged 12 to 36 months, a critical developmental period for children with Down syndrome. This age range was chosen based on evidence highlighting the importance of early intervention in supporting key milestones like socialization and communication, facilitated by heightened brain plasticity during this stage^52,53^. Additionally, the programme addresses a notable gap in the literature regarding the early emergence of adaptive behavior in children under three years old^54,55^.

BabyMICARE follows a standardized manual and consists of 10 weekly sessions conducted by experienced psychologists. It adopts a play-based approach, focusing on skill promotion during free play with objects and aiming for generalization to interactions in daily routines (e.g., eating, toileting). While the primary aims of the program are to increase sensitive responsiveness and reduce directiveness in adult–child interactions, the present study specifically examines its reliability and effectiveness in caregivers with the highest need, meaning those who initially demonstrated greater difficulties in these interactional domains. This targeted approach allows for a focused evaluation of the program’s impact on those requiring the greatest improvement, providing an initial step in assessing its potential benefits. Additionally, the program is also expected to foster broader improvements in dyadic and infant-related dimensions. These may include mutuality, engagement intensity, and positive affect expression, along with infant-specific aspects such as attentiveness to the caregiver and liveliness, which are considered critical for establishing a robust foundation for socio-emotional development. These dimensions could not only serve as indicators of interaction quality but might also act as mediators for key developmental outcomes, such as language acquisition, adaptive skills, and emotional regulation, particularly in children with atypical developmental trajectories^29,30^. Moreover, while this study concentrates on caregivers with the highest need, future research will be necessary to determine whether the program yields similar benefits for caregivers with fewer initial difficulties, ensuring a more comprehensive understanding of its applicability. By targeting these aspects, the program aims to provide a more comprehensive framework for enhancing interaction quality and supporting children’s developmental progress.

## Methods

The study follows a pre-test and post-test design in which participants were assigned according to the characteristics of their interactions to either the control group or the intervention group. The intervention took place over three months, between the baseline and the final evaluation phase.

### Participants

The sample of participants in this intervention study consisted of 40 dyads of infants with Down syndrome and their primary caregivers, which were divided into two groups: the control group (n = 20) and the intervention group (n = 20). Most dyads consisted of mothers and children, both in the control group (n = 17 mothers) and in the intervention group (n = 14 mothers). There is one case in the control group where the primary caregiver is exclusively the father. In addition to the participation of the primary caregiver, in the participating families, another person was allowed to attend the sessions: in some cases, both parents participated (n = 1 in the control group and n = 5 in the intervention group), and in another case, the mother was accompanied by the grandmother (n = 1 in the intervention group). The accompanying adults were not assessed. The choice of the adult that participated in the study was made by the caregivers themselves, according to their availability, considering that there could be no modifications throughout the assessment and intervention process. All the study participants resided in urban areas.

Table 1 reports the sociodemographic data of the study participants. In the intervention group, 45% of participants experienced complications during gestation, while 65% of the control group experienced them. Additionally, 80% of the children in the intervention group and 65% in the control group received medical diagnoses frequently associated with Down syndrome: heart disease; respiratory problems (such as bronchopulmonary dysplasia or obstructive bronchial syndrome); hypothyroidism; West syndrome; hypotonia; swallowing problems; and dysphagia. The percentage of participants who received a prenatal diagnosis of Down syndrome was 25% in the intervention group and 45% in the control group. The rate of kindergarten attendance among participants was low, consistent with national averages in Chile, where 18% of children aged 0–2 years and 47% aged 2–4 years attend kindergarten^56,57^. However, almost all participating children attended early stimulation services, such as speech therapy, physiotherapy, and occupational therapy, starting at an early age. These services, designed to provide targeted stimuli to enhance cognitive, motor, and social development, are crucial for supporting children with neurodevelopmental challenges during the critical early years of life.

**Table 1:** Sociodemographic information of the study participants.

Variables	Intervention Group			Control Group	
Type of participants	Infants	Adults	Infants		Adults
N	20	20	20		20
% Women	35%	95%	55%		90%
Mean (SD) chronological age	20,85 (7,88)	39,85 (4,81)	19 (5,16)		38,95 (4,16)
% Prematurity (< 37 weeks)	10%		15%		
% Pregnancy complications	45%		65%		
% Prenatal diagnosis of Down syndrome	25%		45%		
% Additional diagnostics	80%		65%		
% Kindergarten attendance	20%		15%		
% Early stimulation assistance	100%		90%		
Mean (SD) age of start of early stimulation	3,3 (3,72)		2,2 (1,73)		

*Note.* Chronological age is reported in months for infants and in years for adults. There is one missing value in the control group of the Additional diagnostics variable, so data from 19 dyads are reported. The age of onset of early stimulation is reported in months.

### Recruitment

The sampling procedure was non-probabilistic, voluntary and extended with snowball sampling. Contact was made with nongovernmental organisations working for the social inclusion of people with Down syndrome, who shared information about the research project with the families they serve. A social media campaign was also launched to invite families to participate. Importantly, in Chile, therapeutic support for people with neurodevelopmental conditions is not covered by the public health system, which is why these foundations are especially relevant in their care and attention.

### Procedure

The study was conducted in accordance with the Declaration of Helsinki. All procedures of this study were approved by the Institutional Review Board of the host university. Prior to the start of data collection and programme implementation, informed consent was obtained from caregivers for their participation and that of their children.

The study consisted of three stages, which were conducted in the following order: the preintervention assessment stage, intervention stage and post-intervention assessment stage.

### Preintervention assessment stage

**Fig. 1:**
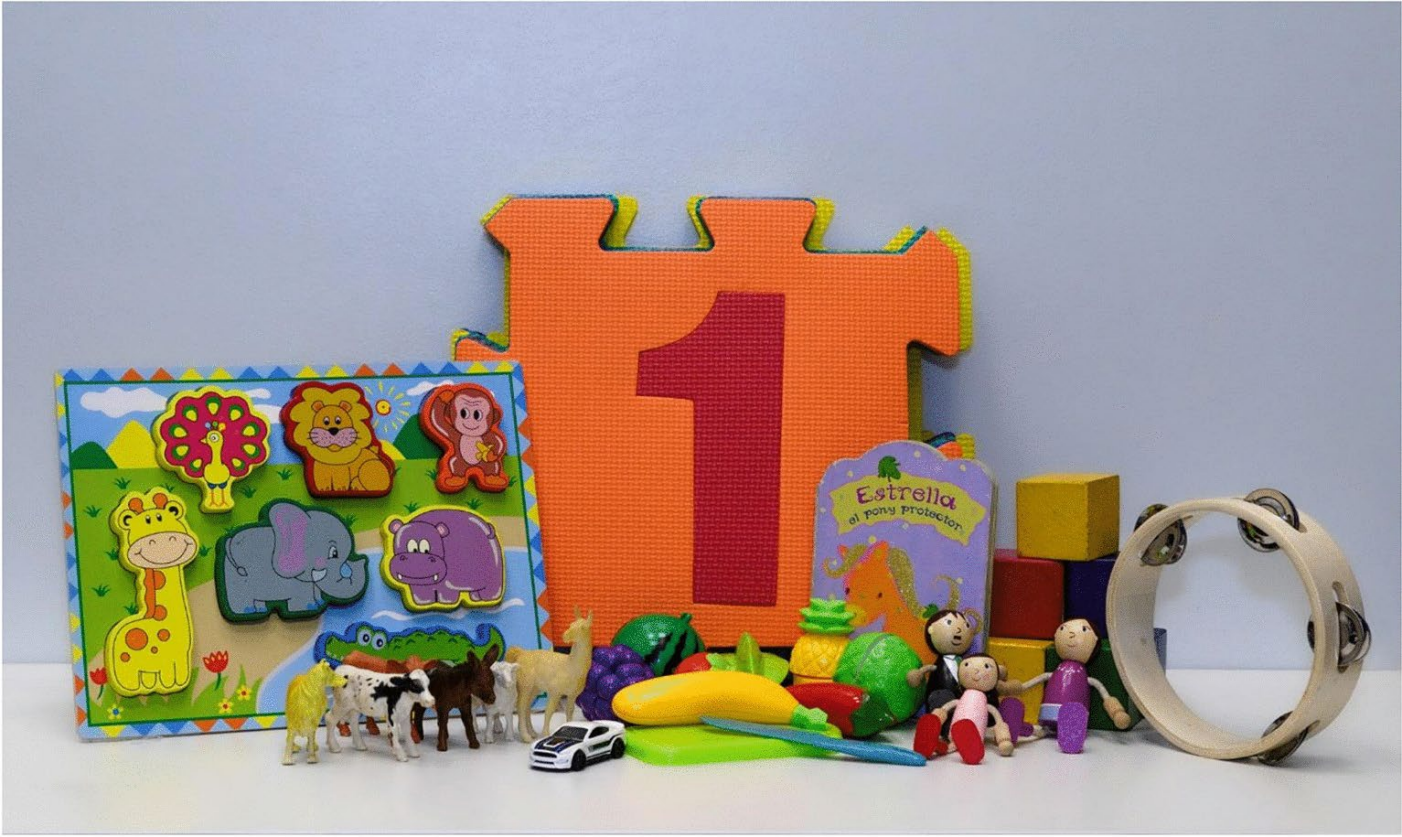
Standardised set of toys used in the study.

The initial assessments were carried out prior to the start of the intervention programme in a daily-life ecological context for the participating families. The duration of each assessment was approximately one hour. During the assessments, questionnaires and tests were administered to assess different aspects of children’s development and their environment. During the same session, caregivers were invited to participate in a free play session with their infants for approximately 10 min. This session was videotaped, and a standardised set of toys and objects was used for all participants. This set included (Fig. 1): 1 EVA rubber mat, 1 wooden puzzle, 8 coloured wooden cubes, 1 book, 1 car, 1 fruit and vegetable set with Velcro with a plastic chopping board and plastic knife, 1 tambourine, 3 wooden dolls (a man, a woman and a girl), and various farm and domestic animal figures.

To preserve the ecological and spontaneous conditions of the interactions, all observations were non-participatory, the evaluators remained silent, still and, if possible, out of sight of the child during the recording. Prior to recording, the only instruction given to caregivers was ‘Play with your child as you normally do’. The play sessions were videotaped by placing the recording devices on a tripod facing the participants to ensure that the angle of the recording allowed the joint actions of the participants to be identified.

The video recordings of caregiver–infant interactions were then coded. For this purpose, the MACI (Manchester Assessment of Caregiver–infant Interaction) system^27^ was used. This is a global assessment measure that identifies the general characteristics of the interaction between an adult caregiver and an infant based on a short video. The scale allows differences in play situations by infants or caregivers to be captured, making its use in this study relevant. Its use is valid for children between 3 and 15 months of age; however, we used this scale for all participants because children with Down syndrome experience developmental delays. This decision follows the procedure published by ^58^ Soukup et al. (2016).

The MACI system consists of 8 scales that receive a score from 1 to 7 each, which allows the evaluator to indicate the intensity at which each of the assessed behaviours appears. Table 2 describes the scales of the system and indicates the participant or reference element to be considered to assess that aspect (caregiver, infant or dyad):

**Table 2:** Scales of the MACI Coding System.

Scale	Reference	Definition
Sensitive responsiveness	Caregiver	Identification of the infant’s signals and response accordingly
Nondirectiveness	Caregiver	Acceptance of the infant’s behaviours or experiences
Attentiveness to Caregiver	Infant	Interest in the caregiver through direct eye contact, joint activity, face/body orientation, imitation, etc
Positive Affect	Infant	Amount and degree of positive expression, vocalisation and body gestures
Negative Affect	Infant	Amount and degree of negative expression, vocalisation and body gestures
Liveliness	Infant	Amount and level of physical activity, in particular behaviours initiated by the infant spontaneously
Mutuality	Dyad	Amount and level of reciprocity and attunement between caregiver and infant
Engagement Intensity	Dyad	Degree of intensity of the interaction at its optimal point. This includes the degree of interest, excitement and positivity

*Note.* The information presented in this table has been extracted from the author’s original publication, which reports on the psychometric properties and validation of the scale (Wan et al., 2017).

The coding of the videos was carried out by professionals from the research team, who had previously received certified training in MACI. The group to which the participants belonged (experimental or control) was hidden from coders. Of the 10 min of total recording, six minutes, usually from minute 1 to minute 7, were coded following the recommendations of the method. In one case, the decision was made to code from minute 3:50 to 9:50 because in the first two minutes of the video, the dyad was not ready and had not started playing. After initial coding by the trained coders, 20% of the pre- and post-intervention recordings were additionally and independently coded to assess the interrater agreement rate. The researchers responsible for the project resolved differences in judgement between the coders when they arose.

Global interrater reliability estimated for the eight MACI variables was good (iota = 0.867). In addition, weighted kappa reliabilities for each of the variables indicated substantial or almost perfect degree of agreement^59^: Sensitive Responsiveness (0.896), Nondirectiveness (0.889), Attentiveness to Caregiver (0.836), Positive Affect (0.885), Negative Affect (0.885), Liveliness (0.803), Mutuality (0.880), and Engagement Intensity (0.818).

The normative study of MACI scale demonstrated solid evidence of reliability and preliminary evidence of validity^27^. Intraclass correlation coefficients (ICCs) for inter-rater reliability ranged from 0.62 (Infant Liveliness) to 0.80 (Caregiver Nondirectiveness at Time 2), indicating good to excellent agreement levels based on^60^ criteria. Regarding evidence of validity in relation to other variables, the study showed convergent relationships: the sensitive responsiveness and infant affect subscales showed moderate correlations with variables related to parental psychological state, such as early postpartum mood (measured by the Edinburgh Postnatal Depression Scale—EPDS)^61^ and perceptions of caregiving received in childhood (measured by the Parental Bonding Instrument—PBI)^62^. Furthermore, the MACI demonstrated discriminant relationships with other measures by differentiating from general dimensions of child development, such as temperament (assessed using the Infant Behavior Questionnaire—Revised)^63^ and language abilities (measured by the Mullen Scales of Early Learning)^64^. These findings support that the MACI is a brief tool for assessing caregiver-infant interactions with good evidence of reliability and validity.

Participants were assigned to the intervention and control groups based on their baseline scores on the sensitive responsiveness and nondirectiveness subscales of the MACI coding system. This decision was guided by both theoretical and practical considerations. These constructs align directly with the primary objective of the intervention, which seeks to enhance caregiver sensitivity and reduce directiveness during interactions. Moreover, evidence from the scientific literature highlights these dimensions as key indicators of high-quality adult-infant interactions, which are critical for fostering positive developmental outcomes^65^. This design was chosen to ensure that the intervention could focus on caregivers with the greatest need for improvement in these critical interaction dimensions, aligning with the program’s objectives. While this approach may limit the comparability between groups, it allowed for a targeted and ethical allocation of resources, providing valuable insights to guide future randomized studies.

From the results of the coding of the pre-intervention measure of the interactions, the scores on the sensitive responsiveness and nondirectiveness scales of all participants were averaged, and the scores were ordered from lowest to highest. The participants in this study were selected from a broader project involving 128 participants (64 with Down syndrome and 64 with typical development). From the group of 64 participants with Down syndrome, the 40 individuals with the lowest scores in parental sensitivity and directiveness were selected for this study. Of these 40 participants, the first 20 (i.e., those with the lowest scores) were assigned to the intervention group, while the remaining 20 were assigned to the control group. This selection strategy reflects the study’s specific objective of assessing the feasibility and effectiveness of the program in caregivers with lower sensitivity and nondirectiveness scores. Future research will be necessary to examine whether the program is similarly effective for caregivers with fewer difficulties, broadening the generalizability of the findings.

The intervention programme, in this case, was applied exclusively in the intervention group. However, the participants in the control group will be offered to receive the intervention in future applications to ensure an ethical implementation of the programme. The caregiver who participated in the sessions was the one who had been in the pre-assessment video. In total, 19 mothers and 1 father were included. However, there were seven families in which two caregivers participated, as described in the sample.

### Intervention stage: BabyMICARE

**Fig. 2:**
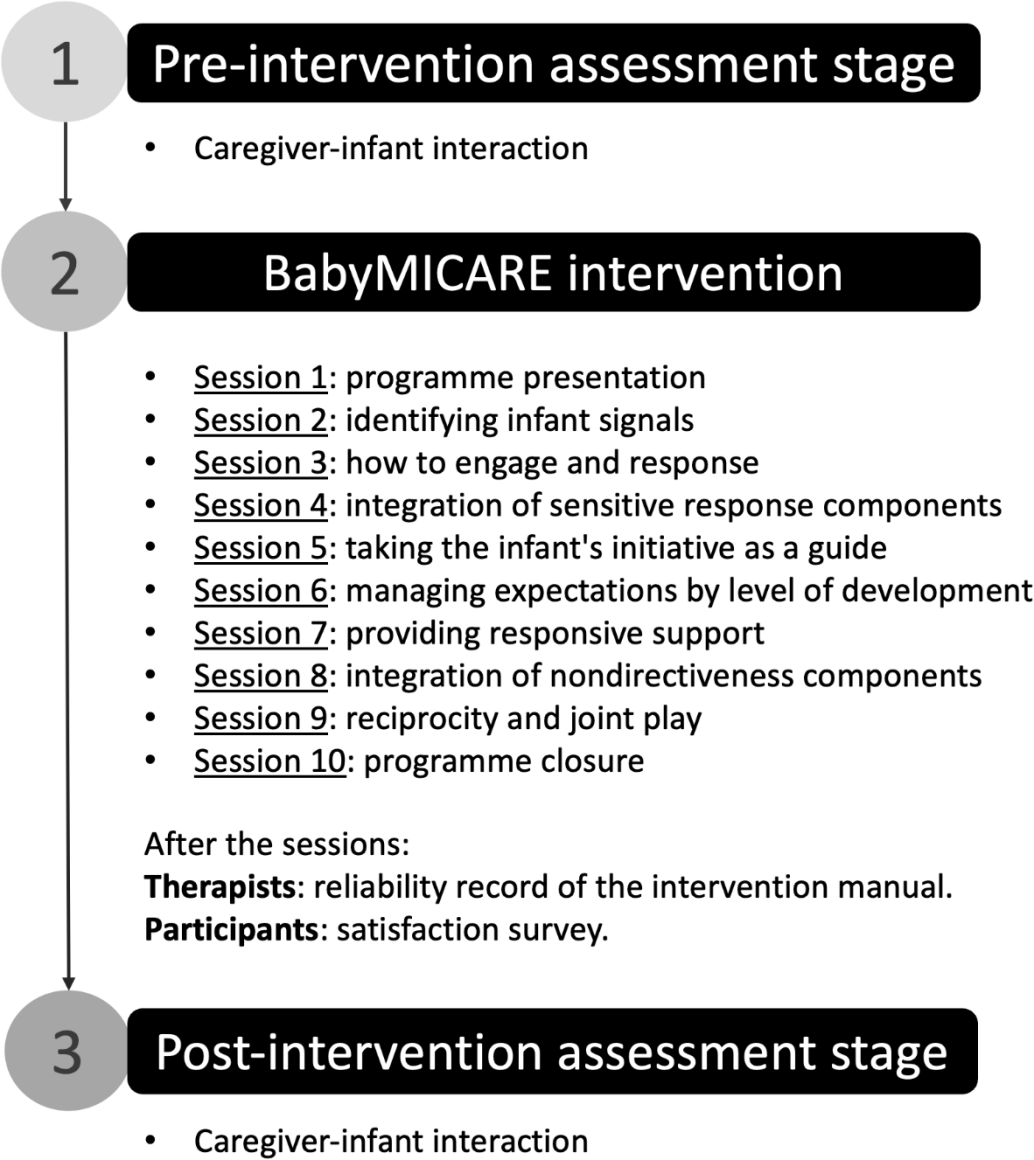
Structure of the BabyMICARE Intervention Program and its assessments.

BabyMICARE is a manualized, play-based intervention programme designed by a team of psychologists and researchers specializing in neurodevelopmental disorders. The programme was implemented by psychologists with master’s degrees and expertise in child neurodevelopment, who were actively involved in the program’s creation from the outset. This involvement provided them with a deep understanding of the objectives and materials for each session. The authors of the programme were supported by research associates, a graphic designer, and transmedia creators. During the implementation, weekly coordination sessions with the BabyMICARE research team ensured proper management of questions and collective decision-making, facilitating the correct and consistent delivery of the intervention. The programme was implemented between August and November 2023. The programme design is summarised in Fig. 2, and the programme manual is freely accessible in the following public repository: https://figshare.com/projects/BabyMICARE/222882

The aim of this intervention was to improve overall interaction quality indicators between caregivers and children with Down syndrome. The BabyMICARE programme is a structured, manualized intervention designed to support the development of children with Down syndrome by enhancing the quality of caregiver-child interactions. The program consists of ten sessions delivered over ten weeks and is designed to meet the specific needs of children and their caregivers. All participants in the study received the intervention following the same structured format, ensuring consistency in delivery and outcomes. The current study had a mixed intervention format, with face-to-face group sessions (Sessions 1 and 10), individual online intervention sessions via video call (Sessions 2 to 9), and self-directed parent review of materials sent via WhatsApp.

Before the first session, a preliminary contact (Session 0) was conducted via a brief telephone call with caregivers. This call served to welcome participants, coordinate the actions to be taken, and provide initial guidance about the program. This preparatory step ensured clarity and alignment between families and therapists before the intervention began.

Sessions 1 and 10 were conducted in person, in a group setting, and lasted approximately 90 min each. These sessions provided families with the opportunity to meet the research team and therapists, ask questions, and connect with other participant families. This collaborative environment fostered a sense of community, enabling families to share experiences, learn from one another, and gain a comprehensive understanding of the program’s objectives. The first session introduced the theoretical foundations and methodology of the intervention, while the final session focused on consolidating learning outcomes, reflecting on progress, and celebrating achievements.

The eight central sessions (Sessions 2 to 9) were delivered individually via video call and lasted 30 to 50 min. These online intervention sessions were focused on developing specific parenting skills through carefully designed activities, tailored to the needs of each family (see Fig. 2). Each session included live, real-time feedback and guidance from the therapist, allowing caregivers to practice skills in their natural context—usually the home. Activities were designed to promote the generalization of skills to daily routines, such as eating and toileting. Therapists employed modeling techniques, using videos to illustrate key behaviors and strategies, and analyzed recorded caregiver-child interactions to provide personalized, evidence-based guidance. It is not recommended to perform these individual sessions in a group format, as this would compromise the personalized accompaniment that is central to the program’s effectiveness.

During the intervention, the therapist provides digital material to the families via WhatsApp, including videos, infographics and simple documents that facilitate the monitoring of the programme and that are sent before or after each scheduled session, according to the material. In this way, participating families can anticipate the contents to be worked on in the session and have documentation that allows them to remember and reinforce what they have learned afterwards. Finally, the therapists completed a manualized fidelity form after each session in which they completed a yes/no question with comments to assess activity implementation and session objectives fulfillment.

### Postintervention assessment stage

The final evaluations, following the completion of the intervention program, were conducted between November and December 2023. Again, home visits were made to the participating families, during which a new videotape of the free play interaction was obtained, of the same duration as in the previous evaluation, and with the same set of toys.

The feasibility of the study was evaluated following the model proposed by Burke et al. (2016) and considering the following variables: (1) Attendance: percentage of sessions attended by each family; (2) Attrition: percentage of participating families who did not complete the program; (3) Rescheduling: percentage of families in which the date of one or more of the program sessions had to be modified; (4) Manualized fidelity: a yes/no question with comments referring to how faithfully the activities were followed and the objectives indicated in the manual for each of the sessions were met; and (5) Family satisfaction: as the sum of the scores of a 7-question questionnaire on caregiver satisfaction with the program, with five response options: Strongly Disagree (1), Disagree (2), Indifferent (3), Agree (4) and Strongly Agree (5); the total score range is 5–35.

On the other hand, the effectiveness of the program was determined through a comparison of the MACI scores of the interactions between the caregiver and the child in the pre- and post-intervention measurements. Specifically, in this study, the scores obtained from the coding of the video recordings, both pre- and post-intervention, on the different MACI scales were used as a measure of comparison.

To assess the feasibility of the program, descriptive analyses of averages and frequencies were performed. For efficacy, pre- and post-intervention repeated-measures ANOVA was used, using the scores obtained on the MACI scale. A correlation analysis was used to assess whether pre-intervention scores may predict post-intervention scores for the intervention group. For the analyses, the full sample of dyads presented in this study (n = 40) was considered. Analyses were performed via JASP^66^, R^67^ and the irr package^68^.

## Results

The results obtained in relation to the feasibility and effectiveness of the BabyMICARE intervention program are presented below.

### Feasibility

The feasibility of BabyMICARE was analysed based on the attendance, attrition and rescheduling percentages of the participants in the different sessions of the program, as well as the fidelity of the therapists’ follow-up of the manual. Similarly, the responses of the participating families regarding their level of satisfaction at the end of the program were analysed. These results are reported in Table 3.

**Table 3:** Results of the feasibility analysis of the BabyMICARE intervention programme.

Variable	Results
% Attendance	100%
% Attrition	0%
% Rescheduling	70%
% Manualized fidelity	97%
Mean Family satisfaction score	34,53

*Note.* The highest possible score for Family Satisfaction is 35.

With respect to participant attendance, all participants attended all sessions of the intervention. Therefore, no participant dropped out of the program.

A significant proportion of participating families (70%) rescheduled one or more sessions during the intervention. The most common reasons for rescheduling included child or caregiver illness, hospitalization, scheduling conflicts preventing the caregiver from arriving on time, or the caregiver’s decision to bring the session forward. On average, each family rescheduled 1.6 sessions, with a total of 32 rescheduled sessions across the 20 families in the intervention group.

Regarding fidelity in the implementation of the intervention by the therapists, the percentage of completion of the activities and fulfilment of the objectives of the sessions was high (97%). In two families, modifications were made in one of the sessions with respect to what was indicated in the manual; in one case, because the activity of daily life (feeding) chosen by the family to work on in session 8 (integration session) could not be carried out, it had to be modified during the session. In the second case, it was necessary to return to the topic worked on in the previous session since the caregiver did not review the information sent prior to the session, so this review had to be included in the activities of the session.

With respect to family satisfaction, an average score of 34.53 was recorded for all participating families, with a maximum score of 35.

### Effectiveness

This section presents the results of the analyses related to the intervention program’s effectiveness. Table 4 displays the mean and standard deviation of pre- and post-intervention scores for each group across all MACI scales. This provides an overview of the descriptive statistics for the outcomes assessed.

**Table 4:** Mean and Standard Deviation of Pre- and Post-Intervention scores for each MACI Scale.

	Intervention group		Control group	
	Pre	Post	Pre	Post
MACI Scales	Mean (SD)	Mean (SD)	Mean (SD)	Mean (SD)
Sensitive responsiveness	2.8 (1.005)	4.850 (1.663)	4.4 (0.94)	4 (1.589)
Nondirectiveness	2.55 (1.317)	4.4 (1.603)	3.9 (0.968)	4.050 (1.638)
Attentiveness to caregiver	4.1 (1.165)	5.2 (1.152)	4.6 (1.188)	4.950 (1.538)
Positive affect	3.6 (1.188)	5 (1.556)	4.150 (1.531)	4.2 (1.735)
Negative affect	2.1 (1.071)	1.8 (1.399)	2 (1.717)	2.25 (1.517)
Liveliness	4.8 (1.642)	5.4 (1.142)	5.05 (1.099)	5.55 (1.191)
Mutuality	3.45 (0.945)	5.05 (1.538)	4.65 (0.875)	4.1 (1.294)
Engagement intensity	3.6 (1.095)	4.65 (1.089)	4.15 (1.04)	4.25 (1.02)

Table 5 reports the results of the efficacy analyses, including within-group and between-group comparisons, highlighting significant changes observed in the intervention group. There were no main effects for Group (all *p* >  = 0.152). At the level of within-subject effects, repeated-measures ANOVA indicated a significant Time effect with medium and large effect sizes for Sensitive Response, Nondirectiveness, Attentiveness to Caregiver, Positive Affect, and Engagement Intensity (all *p* < 0.05, all* η*_*p*_^2^ > 0.116). Significant Time and Group interaction effects with medium to large effect sizes were found for Sensitive Responsiveness, Nondirectiveness, Positive Affect, Mutuality, and Engagement Intensity (all *p* < 0.05, all *η*_*p*_^2^ > 0.102).

**Table 5:** Effectiveness of the BabyMICARE Intervention Programme on each of the MACI Scales*.*

	Main effect for Time		Time x Group interaction effect		Post hoc PRE-POST			
					Control		Intervention	
MACI scales	η_p_^2^	*p*	η_p_^2^	*p*	Cohen’s d	*p*	Cohen’s d	*p*
Sensitive Responsiveness	0,19	< 0,01**	0,341	< 0,001***	0,298	0,584	-1,529	< 0,001***
Nondirectiveness	0,245	< 0,01**	0,19	< 0,05**	-0,107	0,868	-1,315	< 0,001***
Attentiveness to Caregiver	0,149	< 0,05*	0,045	0,191	-0,275	0,769	-0,866	0,053
Positive Affect	0,116	< 0,05*	0,102	< 0,05*	-0,033	0,914	-0,924	< 0,05*
Negative Affect	< 0,001	0,923	0,029	0,29	-0,173	1	0,208	1
Liveliness	0,081	0,076	< 0,001	0,869	-0,388	0,99	-0,466	0,835
Mutuality	0,088	0,063	0,289	< 0,001***	0,461	0,327	-1,341	< 0,01**
Engagement Intensity	0,144	< 0,05*	0,103	< 0,05*	-0,094	0,758	-0,989	< 0,05*

*Note.* * p < 0,05, ** p < 0,01, *** p < 0,001. Only statistically significant results have been highlighted in bold. Following^70^ guidelines , partial eta-squared (η_p_^2^​) values are interpreted as small (η_p_^2^ = 0.01), medium (η_p_^2^ = 0.06), and large (η_p_^2^ = 0.14), while Cohen’s d is categorized as small (d = 0.2), medium (d = 0.5), and large (d = 0.8).

Post hoc analyses between pre- and post-intervention measures for each study group revealed statistically significant improvements, with large effect sizes in the intervention group on five of the eight MACI scales: Sensitive Responsiveness (*p* < 0.001, d = -1.529), Nondirectiveness (*p* < 0.001, d = -1.315), Positive Affect (*p* =  < 0.05, Cohen’s d = -0.924), Mutuality (*p* = 0.001, Cohen’s d = -1.341), and Engagement Intensity (*p* =  < 0.05, d = -0.989). In the control group, no statistically significant change was observed in the pre-post analyses.

Furthermore, as shown in Table 6, there were no significant correlations pre-intervention scores and the magnitude of pre-post changes for each MACI scale in the intervention group.

**Table 6:** Correlations between pre-intervention scores and pre-post changes in the intervention group.

	Intervention group	
MACI Scales	r	p
Sensitive responsiveness	0.170	0.474
Nondirectiveness	0.289	0.216
Attentiveness to caregiver	0.063	0.793
Positive affect	-0.142	0.549
Negative affect	0.014	0.953
Liveliness	-0.123	0.604
Mutuality	-0.053	0.826
Engagement intensity	-0.123	0.604

## Discussion

The main purpose of this article is to present results on the feasibility and effectiveness of BabyMICARE, a play-based manualised program designed to improve parenting skills and the interactions between caregivers and their infants with Down syndrome. With respect to the feasibility of the program, we observed high rates of attendance and continuity of participants during the sessions, along with ease on the part of the therapists to follow the manualised program and excellent evaluation by the families.

During the implementation of the BabyMICARE program, session rescheduling was frequently required due to participants’ limited availability, often because of children’s medical appointments or caregivers’ work commitments. This flexibility proved essential in accommodating the unique demands on families of children with Down syndrome or other developmental vulnerabilities, ensuring no attrition and demonstrating the program’s feasibility in the Chilean context, where such family accompaniment is not commonly offered by public health or private foundations.

While the program’s flexibility supports adherence, the frequent need for rescheduling underscores a key challenge. Medical appointments remain a top priority for these families, and without this adaptability, attrition rates would likely increase. The low attrition rate observed in our study suggests that the motivation of families to participate in such interventions is high, despite the unforeseen difficulties that may arise. Families are not withdrawing from the study in the face of challenges; instead, they actively strive to continue their participation. Future implementations could explore additional solutions, such as asynchronous resources or more dynamic scheduling systems, to further mitigate scheduling conflicts and enhance accessibility without compromising participation.

With respect to the effectiveness of the program, the significant improvements in scores on many of the MACI scales (sensitive responsiveness, nondirectiveness, positive affect, mutuality, and engagement intensity) demonstrate the success of the program in improving parent–child interactions quality. However, given that the study focused on participants with the highest need, these findings apply specifically to caregivers who initially showed greater difficulties in these interactional domains. While the absence of significant correlations between pre-intervention scores and pre-post changes within the intervention group suggests that individual differences in baseline levels did not systematically predict improvement, this result should be interpreted within the context of the selected sample. The study was not designed to assess whether the program is equally effective across the full spectrum of caregivers, and future research is needed to determine its impact on those with fewer initial difficulties. Notably, most of the MACI scales in which significant improvements were obtained are directly related to adult behaviors or global interaction (except for the positive affect scale). This highlights the program’s capacity to foster meaningful improvements within the highest-need group, reinforcing its relevance for families requiring greater support in these interactional dimensions.

Notably, no significant improvements were observed in our study in other scales directly related to infants, such as Attentiveness to Caregiver. While a recent meta-analysis on joint attention skills in infants with Down syndrome suggested that their competence level is comparable to that of typically developing peers^69^, and that their social engagement may provide important opportunities for stimulation and learning, this hypothesis is not fully supported in light of contradictory findings from^32^. The latter study detected significant differences in Attentiveness to Caregiver between infants with Down syndrome and their typically developing counterparts, suggesting that the baseline competence of infants with Down syndrome might not always be sufficient to sustain adequate engagement.

Furthermore, it is reasonable to argue that the lack of improvement in this domain may be due to the program’s primary focus on enhancing caregiver skills, rather than directly targeting infant behaviors. This alternative explanation aligns with the program’s objectives and highlights the importance of prioritizing caregiver strategies to support infant development indirectly.

As the results show, BabyMICARE also fostered a significant change in the reciprocal interaction between caregivers and infants. By creating spaces and opportunities for families to play and enjoy freely with their infants, the emphasis on reaching developmental milestones is temporarily set aside, allowing for more natural and enriching interactions that provide valuable experiences for the infant, a change that can be developmentally beneficial. This playful approach not only benefits the well-being of caregivers and infants but also allows for the integration of daily routines into play sessions, offering an innovative format for the improving interactions that may support the development of adaptive behaviours in infants.

We also emphasize the importance of developing BabyMICARE locally, within the Latin American context, as a key contribution of the intervention. This regional focus ensures cultural relevance by addressing the unique social, economic, and familial dynamics of the region. Tailoring the program to local caregiving practices, available resources, and community values enhances its effectiveness. Additionally, in many Latin American countries, including Chile, childcare services and early intervention programs are often insufficiently covered by public systems. Developing BabyMICARE locally helps fill this gap, providing families with accessible and contextually appropriate support. This approach also enables the program to address specific challenges, such as cultural nuances in caregiver-child interactions and disparities in access to resources, aligning with global calls for equity in early intervention and serving as a model for culturally adaptive approaches in similar contexts.

Although the results are successful and promising, the present study has several limitations worth mentioning. The sample size is relatively small and limited to two specific regions of Chile, which may affect the generalizability of the results. In addition, the absence of follow-up assessments makes it difficult to evaluate the programme’s results in the long term.

A key limitation of this study is the non-randomized assignment of participants, which may have influenced the findings by reducing the comparability between the intervention and control groups. However, this design allowed us to prioritize caregivers with the greatest need for improvement in critical interaction dimensions, aligning with the program’s objectives. Future studies could build on these findings by employing randomized designs to further explore the program’s effectiveness across a broader range of participants.

Future research could focus on expanding the sample size and including participants from different regions and sociocultural contexts to increase the generalizability of the results. In addition, further evaluations could be explored to measure the long-term impact of the program on children’s development and family dynamics. Adaptations of the program could also be made for different groups at risk or with different specific needs, such as premature infants, or with vulnerability to autism. Finally, new studies could be conducted with the aim of comparing scores not only on the characteristics of interactions but also on infants’ level of adaptive behaviour.

The findings of this study highlight the program’s potential to significantly benefit families by improving caregivers’ interactions with their children in areas critical to fostering healthy development. However, given that the study focused on caregivers with the highest need, these findings specifically apply to families whose initial interactions were characterized by greater difficulties in sensitivity and nondirectiveness. While the analysis did not reveal significant associations between baseline scores and improvements within the intervention group, this should not be interpreted as evidence that the program is equally effective across all caregivers, as its impact on those with fewer initial difficulties remains untested. While the non-randomized design and targeted allocation of participants limit causal claims about the program’s general effectiveness, the observed improvements underscore its overall value in enhancing parent–child interactions.

In conclusion, BabyMICARE is presented as a viable and effective intervention for improving the interactions between caregivers and infants and toddlers with Down syndrome. This programme offers an innovative approach that not only provides practical support for caregivers but also fosters an environment of play and enjoyment, which may ultimately benefit the development of adaptive behaviours in children and, with it, their autonomy and social participation. These promising results provide a strong foundation for future research to further validate and expand the reach of this intervention.

## Data Availability

The datasets generated and analysed during the current study are available from the corresponding author on reasonable request.
